# Towards a consensus regarding global signal regression for resting state functional connectivity MRI

**DOI:** 10.1016/j.neuroimage.2016.11.052

**Published:** 2017-07-01

**Authors:** Kevin Murphy, Michael D. Fox

**Affiliations:** a Cardiff University Brain Research Imaging Centre (CUBRIC), School of Psychology, Cardiff University, Cardiff, CF24 4HQ, United Kingdom; b Cardiff University Brain Research Imaging Centre (CUBRIC), School of Physics and Astronomy, Cardiff University, Cardiff, CF24 4HQ, United Kingdom; c Berenson-Allen Center for Noninvasive Brain Stimulation, Department of Neurology, Beth Israel Deaconess Medical Center, Harvard Medical School, Boston, MA, United States; d Department of Neurology, Massachusetts General Hospital, Harvard Medical School, Boston, MA, United States; e Athinoula A. Martinos Center for Biomedical Imaging, Harvard Medical School, Boston, MA, United States

## Abstract

The number of resting state functional connectivity MRI studies continues to expand at a rapid rate along with the options for data processing. Of the processing options, few have generated as much controversy as global signal regression and the subsequent observation of negative correlations (anti-correlations). This debate has motivated new processing strategies and advancement in the field, but has also generated significant confusion and contradictory guidelines. In this article, we work towards a consensus regarding global signal regression. We highlight several points of agreement including the fact that there is not a single “right” way to process resting state data that reveals the “true” nature of the brain. Although further work is needed, different processing approaches likely reveal complementary insights about the brain's functional organisation.

## Highlights

• Global signal regression is a controversial resting state fMRI pre-processing option.
• The debate about GSR has generated significant confusion and contradictory guidelines.
• Here, we present our consensus statement on the use of GSR in resting state analyses.
• There is no “right” way to pre-process that reveals the “true” nature of the brain.
• Different processing approaches reveal complimentary insights about brain function.

## 1 Introduction

The global signal in neuroimaging can be defined as the time series of signal intensity averaged across all brain voxels. Because most imaging studies are interested in region-specific changes in brain activity and because non-neuronal sources can contribute to the global signal (Birn et al., 2006, Glover et al., 2000, Wise et al., 2004), various methods have been used to correct for global signal changes. Global signal regression (GSR) is the removal of the global signal from the time series of each voxel through linear regression. This procedure was originally developed for and applied to task-based fMRI data (Aguirre et al., 1998, Macey et al., 2004, Zarahn et al., 1997). However its greatest use, and greatest controversy, has come with the advent of resting state functional connectivity MRI.

A landmark study in 2005, building upon earlier work (Fransson, 2005, Greicius et al., 2003), used resting fMRI data to show that the brain was organised into two diametrically opposed, widely distributed networks (Fox et al., 2005). Spontaneous fluctuations in the default mode network were anti-correlated (negatively correlated) with fluctuations in the task positive network. This formed an appealing narrative as these networks were routinely modulated in opposite directions by task paradigms (Fox et al., 2005, Raichle et al., 2001). However, the impact of global signal regression on these anti-correlated networks was not addressed.

In 2009, two papers examined this issue, but gave opposite recommendations as to whether GSR should be used in the processing of resting state functional connectivity data (Fox et al., 2009, Murphy et al., 2009). Murphy et al. was the first to show that GSR mathematically mandates the presence of anti-correlations. Because anti-correlations following GSR could be an artefact of the processing technique, Murphy et al. concluded that GSR should not be used. Fox et al. replicated these results including the mathematical mandate; however also found that several characteristics of anti-correlated networks could not be attributed to GSR. Because GSR enhanced the detection of system-specific correlations and improved the correspondence between resting-state correlations and anatomy, they concluded that GSR can be beneficial (Fox et al., 2009).

Since this time many studies have tried to determine whether GSR is beneficial or detrimental for processing and interpreting resting state functional connectivity data. Several alternatives to GSR that attempt to correct for global variance but avoid the “mathematical mandate” have also been proposed. However, often the point of the argument is missed; it is about how to interpret correlation values after pre-processing in a particular way. Without an accepted gold standard, this literature has continued to produce contradictory conclusions and recommendations. Researchers are left in a difficult position regarding whether to utilize GSR or not. As authors of two of the early papers that came to conflicting conclusions, we have chosen to work together to review the data relevant to this question, highlight points of agreement, and come to a consensus regarding GSR.

## 2 Global Signal Regression

### 2.1 What is the global signal?

Resting state correlation distributions are heavily skewed towards positive values when no pre-processing is performed (Chai et al., 2011, Fox et al., 2009, He and Liu, 2011, Keller et al., 2013, Murphy et al., 2009). Furthermore, nearly all voxels show statistically significant correlation with the global signal (Fox et al., 2009). This suggests that there is some component in the timeseries that is common to all voxels, thus is global. Whether such a global signal arises from common neural fluctuations or from fluctuations in a confounder that has a global effect (e.g. arterial CO_2_) is difficult if not impossible to assess based on fMRI data alone.

The fMRI signal is based on the blood oxygenation level dependent (BOLD) contrast. Rather than being a direct measure of neural activity, BOLD signals are a complex interaction of metabolism (CMRO2), blood flow (CBF) and blood volume (CBV). Any phenomenon that affects the balance between these 3 parameters will cause changes in resting-state BOLD signals that may be spuriously correlated across regions. Many resting-state fMRI confounds are global in nature, arising from motion, cardiac and respiratory cycles, arterial CO_2_ concentration, blood pressure/cerebral autoregulation and vasomotion (Murphy et al., 2013). Variance related to these processes will be reflected in the global signal; for example, low-frequency respiratory volume and cardiac rate regressors display significant shared variance with the global signal (Chang and Glover, 2009), as do motion parameters (Power et al., 2014). GSR reduces BOLD spectral power, with Zhu and colleagues concluding that a large portion of resting signals can be attributed to the vascular effects (Zhu et al., 2015). Therefore, removal of the global signal variance from all voxel timeseries using regression (i.e., GSR), can, at least partially, remove these unwanted global confounds.

However, in addition to these non-neuronal confounds, the global signal also includes fluctuations in neuronal activity. When noise signals are low, the global signal resembles the time course of the largest cluster in the brain, which in real data is the DMN (Chen et al., 2012). However, when noise signals are high, this may not be the case (Fox et al., 2009). Comparisons with spontaneous fluctuations in LFPs from a single cortical site in monkeys show positive correlation with fMRI signals over nearly the entire cerebral cortex, demonstrating that the global signal is tightly coupled to underlying neural activity (Schoelvinck et al., 2010). Further evidence that the global signal has a neural component is demonstrated by the negative correlation between its amplitude and EEG vigilance measures across subjects (Wong et al., 2013). Changes in the global signal amplitude between an eyes-open and eyes-closed condition are associated with changes in EEG vigilance (Wong et al., 2015). Ingestion of caffeine significantly decreases global signal amplitude and increases EEG measures of vigilance (Wong et al., 2012). Furthermore, physiological effects such as changes in arterial CO_2_ can influence neural activity globally (Driver et al., 2016).

Therefore, although the global signal reflects non-neuronal confounds in the data, it also likely includes a neuronal component whose relative contribution may be dependent on the brain region and dataset in question (Fox et al., 2009, Wong et al., 2012). Whether removing this signal through GSR is good or bad depends on the scientific question and must be considered when interpreting the results. How global neural components interact with focal resting neural activity should be investigated for a full understanding of brain function.

### 2.2 What does GSR do?

GSR uses linear regression to remove shared variance between the global signal and the time course of each individual voxel. Mathematic details regarding the precise algebraic operation performed by GSR and the resulting effect of GSR on residual correlation values has been published previously (Fox et al., 2009, Murphy et al., 2009). The algebraic consequence of GSR is that, for any seed, the mean value of voxel-on-seed beta coefficients over the whole brain is *exactly* zero and the distribution of Pearson correlations over the brain becomes *approximately* zero centered. In other words, GSR shifts the distribution of functional connectivity values from being predominantly positive to both positive and negative in any given subject. To the extent that this shift shares similar spatial topography across subjects, these negative correlations can appear as significant anti-correlations in group-level analyses.

### 2.3 Are there benefits to global signal regression?

Global signal regression can improve the specificity of positive correlations (Fox et al., 2009, Weissenbacher et al., 2009), improve the correspondence to anatomical connectivity (Fox et al., 2009), and help remove non-neuronal sources of global variance such as respiration (Birn, 2012) and movement (Power et al., 2014, Yan et al., 2013). As such, there is a reasonable motivation for using GSR as a pre-processing technique. However, this does not mean that GSR is the best or only approach for achieving these benefits. For example, one study found that anatomical specificity was better using alternatives to GSR (Chai et al., 2011). Similarly, GSR may make correlation estimates more susceptible to motion (Jo et al., 2013) and treating head motion as a global confound may not be appropriate (Spisak et al., 2014).

The impact of GSR on test-retest reliability appears to be mixed. Including GSR reduced test-retest reliability in older adults (Guo et al., 2012) and showed a lower proportion of reliable connections in both young and old groups (Song et al., 2012). However GSR increased consistency of functional connections within-subject across scans (Song et al., 2012) and had a negligible effect on the temporal reliability in the language network (Zhu et al., 2014). Test-retest reliability of graph theoretical measures can be decreased (Liang et al., 2012), increased (Braun et al., 2011), or unaffected by GSR (Andellini et al., 2015). Reliability of ReHo measures is reportedly decreased (Zuo et al., 2012). Finally, using GSR on arterial spin labelling data improves temporal SNR and test-retest stability of CBF measurements (Wang, 2012).

Whether GSR helps or hurts detection of brain behaviour relationships has also depended on the study. Including GSR led to stronger relationships between connectivity and cognitive function (Hampson et al., 2010), helped identify face sensitive areas within the FFA (Kruschwitz et al., 2015) and was useful in predicting symptoms from focal brain lesions (Boes et al., 2015). In contrast, GSR hurt the ability to find relationships between connectivity and behaviour in Autism Spectrum Disorder (Gotts et al., 2013).

It should be noted that, although GSR is mainly used as a means for reducing artefacts, there are cases where GSR may be useful for other reasons. Zero-centring is often used as a strategy in analyses of correlated variables in other areas of research, for example, in the study of gene co-expression patterns (Langfelder and Horvath, 2007).

### 2.4 Can global signal regression introduce spurious anti-correlations?

Multiple modelling studies have shown that global signal regression can introduce “artefactual” anti-correlations that were not originally present in the modelled data (Anderson et al., 2010, Murphy et al., 2009, Saad et al., 2012). Anderson et al. demonstrated that anti-correlations are introduced during GSR for any two networks as a linear function of their size (Anderson et al., 2010). Simulating a group comparison study using 3 ROIs, Saad et al. showed that GSR alters local and long-range correlations, leading to group differences in regions that were not modelled to have any (Saad et al., 2012). The degree to which these modelling results using a small number of regions applies to human BOLD data with presumably higher dimensionality is unclear.

### 2.5 Does global signal regression introduce spurious anti-correlations in real data?

Although spurious anti-correlations are clearly present in modelling studies, it is unclear which, if any, anti-correlations observed in human fMRI data are also spurious. This question is difficult to answer as there is not a clear gold standard with which to compare results. As such, studies that have tried to address this have often come to different conclusions. Anti-correlation with the orbits when the soft tissue is included in the global signal is taken as evidence that artefactual anti-correlation can be present in human data (Anderson et al., 2010). Similarly, several studies have observed anti-correlations only when GSR is applied (Ibinson et al., 2015, Weissenbacher et al., 2009). However other studies have found anti-correlations without GSR, suggesting that they may not depend on this pre-processing step. For example, anti-correlations can be seen with physiological noise correction (Chang and Glover, 2009), component based noise reduction (Chai et al., 2011), or ingestion of caffeine (Wong et al., 2013).

Studies have tried to determine the existence of anti-correlated networks with complementary techniques. Both positive and negative correlations were shown to have neurophysiological correlates as measured by ECoG (Keller et al., 2013). However, GSR resulted in some BOLD anti-correlations that were not present in the ECoG data. Anti-correlations in a well characterised frontolimbic circuit between infralimbic cortex and amygdale were observed in awake rats both with and without GSR, however the relationship was absent in anaesthetised rats even after GSR (Liang et al., 2011). The anaesthesia weakened the positive correlations and abolished the negative correlations suggesting that the anti-correlations found in the awake rats were not solely due to the GSR pre-processing step.

### 2.6 How does GSR influence other resting state measures?

The effects of GSR on graph theoretical measures and regional homogeneity have been investigated; however without a gold-standard it is difficult to know whether these effects are positive or negative. Significant differences in global network metrics (Liang et al., 2012) and local graph metrics (Borchardt et al., 2016) can be impacted by GSR. When GSR is implemented, heritability estimates of graph theoretical measure (mean clustering coefficient, modularity, rich-club coefficient, global efficiency, small-worldness) are substantially reduced (Sinclair et al., 2015). Since GSR alters network topology in the left histogram tail (most negative correlations) with clustering coefficient and assortativity converging to zero, networks constructed from the absolute value of the correlations coefficient are compromised following GSR (Schwarz and McGonigle, 2010). ReHo values are reduced by GSR but the spatial distribution is unchanged (Qing et al., 2013). Reproducible differences in ReHo between eyes open and eyes closed conditions exist in areas that differ depending on whether GSR was used or not.

### 2.7 Are there alternatives to GSR?

Given that removal of global sources of variance seems to have some value, but GSR itself introduces interpretive complexity, many alternatives to GSR have been proposed that circumvent that “mathematical mandate”. These techniques aim to remove common fluctuations that arise from uninteresting sources, such as physiological noise, to better focus on fluctuations related to region-specific changes in neural activity. When using these techniques, it is important to remember that all pre-processing approaches change the resulting correlation structure to some extent and it's difficult to know whether this is beneficial or detrimental.One technique is to record physiological signals simultaneously to acquiring resting state fMRI data, and then remove the related variance (Birn, 2012, Murphy et al., 2013). Motion confounds can be estimated and removed from the data itself (Friston et al., 1996). Cardiac and respiratory noise can be removed using techniques such as RETROICOR (Glover et al., 2000), RVT (Birn et al., 2006), heart rate (Shmueli et al., 2007) and end-tidal CO_2_ correction (Murphy et al., 2013, Wise et al., 2004).

When physiological data is not recorded alongside MRI data, there are still alternatives to GSR that can be performed using the MRI data alone. One can regress out timecourses based on non-grey matter signals (Behzadi et al., 2007, Chai et al., 2011, Weissenbacher et al., 2009) or decompose the data into signal and noise components using ICA (Griffanti et al., 2014, Perlbarg et al., 2007). Similarly, one can use partial correlation between different regions to remove sources of shared variance (Zhang et al., 2008). Using the global signal itself to estimate subject-specific respiratory and cardiac response functions has also been proposed (Falahpour et al., 2013).

More complex data-based alternatives to GSR have also been proposed. A random subspace method for functional connectivity (RSMFC) was developed that estimated partial correlation between a seed region and each target brain voxel using multiple subsets of randomly sample voxels (Chen et al., 2013). A data-driven noise correction method termed APPLECOR (Affine Parameterization of Physiological Large-scale Error CORrection) models spatially-common physiological noise as a linear combination of an additive term and a mean-dependent multiplicative term (Marx et al., 2012). A method based on the phase-shifting of soft tissue signals, such as those from the eyes, (PSTcor) was proposed (Anderson et al., 2010). These complex techniques all have some theoretical and practical advantages compared to GSR and can produce different results. However, they also have a downside, namely that their effect on the data is less intuitive and less investigated compared to the simpler GSR method.

Other methods have tried to determine when GSR is suitable. A measure was developed, entitled the Impact of the Global Average on Functional Connectivity (IGAFC), that provides a threshold at which the impact of regressing-out the global signal would be large enough to introduce spurious anti-correlations (Carbonell et al., 2013). A complementary study showed that an adaptive thresholding of correlation values improves reliability, mainly by accounting for the global signal variance (Gorgolewski et al., 2012). Using a framework to characterise the properties of the global signal, it was demonstrated that a proportion of the global signal can be viewed as an additive confound that increases with mean BOLD amplitude, therefore can be minimised (He and Liu, 2011).

Recently, many reviewers encourage repeating analyses with and without GSR, to ensure that results (especially anti-correlations) are not due solely to GSR pre-processing. In general, this practice can aid in result interpretation. However, results using alternative approaches for removing the global signal are usually quite similar (Boes et al., 2015, Chai et al., 2011), while results that use no correction for global signal fluctuations can be quite different (Fox et al., 2009, Gotts et al., 2013, Murphy et al., 2009, Saad et al., 2012, Wong et al., 2012). In this latter case, a failure to reproduce results without some type of global signal correction does not mean the results are an “artefact”. Rather, it means that some correction for global signal fluctuations was necessary to produce the finding of interest, and attempts to replicate the finding may also benefit from global signal correction.

## 3 Points of agreement

### 3.1 Should I use global signal regression?

Different processing techniques likely produce different complementary insights into the brain's functional organization, none of which has a monopoly on truth. For example, if one is trying to predict the response of different brain regions to a task, GSR appears useful as it correctly predicts the spatial distribution of relative increases or decreases in activity. However if one is trying to predict the electrophysiological relationship between regions measured with implanted electrodes in the setting of large global (and neuronal) fluctuations in arousal, analyzing the data without global signal regression may prove more accurate. The “correct” approach is the one that proves most useful for predicting the feature of interest.

A simple analogy is analysing the ripples on a pond on a windy day. If the goal is to determine which direction the wind is blowing, one needs to analyse the data with global fluctuations included. If one is trying to determine the location of a small pebble thrown into the pond, regressing out common fluctuations may be critical. Neither analysis is a more accurate representation of the “true” nature of the pond. Rather, if applied and interpreted correctly, they provide complementary information.

Another, perhaps closer analogy comes from analysis of EEG data routinely used in clinical practice to detect and localize seizures. EEG potentials can be displayed with respect to an average reference (i.e. the global signal), but can also be displayed with respect to neighbouring electrodes or even a reference electrode attached to the ear. The “correct” EEG montage is the one the best allows a clinician to see an epileptiform discharge, and EEG data is often viewed in multiple different ways to best achieve this goal.

### 3.2 Consensus statements and recommendations

Here, we have attempted to produce some consensus statements and recommendations to help researchers decide if GSR is appropriate in the context of their specific experimental hypotheses.

1. Correction for global signal fluctuations with any technique including GSR has a significant impact on resting state functional connectivity results. Methods must be clearly described and results interpreted in the context of the method applied.
2. The mathematics of global signal regression (GSR) mandate that functional connectivity analyses performed using this processing step show both positive and negative values that average to zero across all voxels in a single subject.
3. GSR can introduce “artefactual” anti-correlations into simulated data that did not originally consist of modelled anti-correlations. By extension, anti-correlations observed in human fcMRI data after GSR could be “artefactual” in a similar sense. Whether certain human fcMRI anti-correlations ARE “artefactual” in this sense is a separate question and difficult to test without a “gold standard”.
4. The mathematics of GSR does not mandate the specific spatial distribution of anti-correlation, the consistency of anti-correlations across a group, or the existence of statistically significant anti-correlations after group-level random effects analysis. As such, there are examples where resting state connectivity data processed with GSR do not show significant anti-correlations.
5. Several advantages of GSR have been reported including closer relationship to DTI-based anatomy, better delineation of subcortical nuclei, improved specificity of positive correlations, and removal of motion, cardiac and respiratory signals known to correlate with the global signal.
6. Several alternatives to GSR have been proposed that technically avoid the above “mathematical mandate” whilst aiming to remove the uninteresting contributions to common fluctuations. However, all pre-processing methods change the correlation structure to some extent. Technically avoiding the mathematical mandate does not mean that interpretation problems are avoided.
7. The global signal is composed of both neural and non-neural signals. This fact should be taken into account when interpreting results.
8. GSR should be used with care when comparing groups with different noise characteristics or varying neural network structures. GSR can have differential effects on the groups, removing noise with varying success or spreading nodal changes throughout the entire network.

### 3.3 Summary

In summary, including global signal regression in the processing of resting state functional connectivity data is not inherently right or wrong. Whether GSR is a useful processing step likely depends on the scientific question one seeks to address. Results must be interpreted properly within the context of the pre-processing method applied and different methods may provide complementary insights into the brain's functional organization.

## References

[bib1] Aguirre G.K., Zarahn E., D'Esposito M. (1998). The inferential impact of global signal covariates in functional neuroimaging analyses. NeuroImage.

[bib2] Andellini M., CannatÃ V., Gazzellini S., Bernardi B., Napolitano A. (2015). Test-retest reliability of graph metrics of resting state MRI functional brain networks: a review. J. Neurosci. Methods.

[bib3] Anderson J.S., Druzgal T.J., Lopez-Larson M., Jeong E.K., Desai K., Yurgelun-Todd D. (2010). Network anticorrelations, global regression, and phase-shifted soft tissue correction. Hum. Brain Mapp..

[bib4] Behzadi Y., Restom K., Liau J., Liu T.T. (2007). A component based noise correction method (CompCor) for BOLD and perfusion based fMRI. NeuroImage.

[bib5] Birn R.M. (2012). The role of physiological noise in resting-state functional connectivity. NeuroImage.

[bib6] Birn R.M., Diamond J.B., Smith M.A., Bandettini P.A. (2006). Separating respiratory-variation-related fluctuations from neuronal-activity-related fluctuations in fMRI. NeuroImage.

[bib7] Boes A.D., Prasad S., Liu H., Liu Q., Pascual-Leone A., Caviness V.S., Fox M.D. (2015). Network localization of neurological symptoms from focal brain lesions. Brain.

[bib8] Borchardt V., Lord A.R., Li M., van der Meer J., Heinze H.J., Bogerts B., Breakspear M., Walter M. (2016). Preprocessing strategy influences graph-based exploration of altered functional networks in major depression. Hum. Brain Mapp..

[bib9] Braun U., Plichta M.M., Esslinger C., Sauer C., Haddad L., Grimm O., Mier D., Mohnke S., Heinz A., Erk S., Walter H., Seiferth N., Kirsch P., Meyer-Lindenberg A. (2011). Test-retest reliability of resting-state connectivity network characteristics using fMRI and graph theoretical measures. NeuroImage.

[bib10] Carbonell F., Bellec P., Shmuel A. (2013). Quantification of the impact of a confounding variable on functional connectivity confirms anti-correlated networks in the resting-state. NeuroImage.

[bib11] Chai X.J., Castanan A.N., Ongur D., Whitfield-Gabrieli S. (2011). Anticorrelations in resting state networks without global signal regression. NeuroImage.

[bib12] Chang C., Glover G.H. (2009). Effects of model-based physiological noise correction on default mode network anti-correlations and correlations. NeuroImage.

[bib13] Chen G., Xie C., Ward B.D., Li W., Antuono P., Li S.J. (2012). A method to determine the necessity for global signal regression in resting-state fMRI studies. Magn. Reson. Med..

[bib14] Chen T., Ryali S., Qin S., Menon V. (2013). Estimation of resting-state functional connectivity using random subspace based partial correlation: a novel method for reducing global artifacts. NeuroImage.

[bib15] Driver I., Whittaker J.R., Bright M.G., Murphy K. (2016). Arterial CO_2_ fluctuations modulate neuronal rhythmicity: implications for MEG and fMRI studies of resting state networks. J. Neurosci..

[bib16] Falahpour M., Refai H., Bodurka J. (2013). Subject specific BOLD fMRI respiratory and cardiac response functions obtained from global signal. NeuroImage.

[bib17] Fox M.D., Snyder A.Z., Vincent J.L., Corbetta M., Van Essen D.C., Raichle M.E. (2005). The human brain is intrinsically organized into dynamic, anticorrelated functional networks. Proc. Natl. Acad. Sci. USA.

[bib18] Fox M.D., Zhang D., Snyder A.Z., Raichle M.E. (2009). The global signal and observed anticorrelated resting state brain networks. J. Neurophysiol..

[bib19] Fransson P. (2005). Spontaneous low-frequency BOLD signal fluctuations: an fMRI investigation of the resting-state default mode of brain function hypothesis. Hum. Brain Mapp..

[bib20] Friston K.J., Williams S., Howard R., Frackowiak R.S., Turner R. (1996). Movement-related effects in fMRI time-series. Magn. Reson. Med..

[bib21] Glover G.H., Li T.Q., Ress D. (2000). Image-based method for retrospective correction of physiological motion effects in fMRI: retroicor. Magn. Reson. Med..

[bib22] Gorgolewski K.J., Storkey A.J., Bastin M.E., Pernet C.R. (2012). Adaptive thresholding for reliable topological inference in single subject fMRI analysis. Front. Hum. Neurosci..

[bib23] Gotts S.J., Saad Z.S., Jo H.J., Wallace G.L., Cox R.W., Martin A. (2013). The perils of global signal regression for group comparisons: a case study of Autism Spectrum Disorders. Front. Hum. Neurosci..

[bib24] Greicius M.D., Krasnow B., Reiss A.L., Menon V. (2003). Functional connectivity in the resting brain: a network analysis of the default mode hypothesis. Proc. Natl. Acad. Sci. USA.

[bib25] Griffanti L., Salimi-Khorshidi G., Beckmann C.F., Auerbach E.J., Douaud G., Sexton C.E., Zsoldos E., Ebmeier K.P., Filippini N., Mackay C.E., Moeller S., Xu J., Yacoub E., Baselli G., Ugurbil K., Miller K.L., Smith S.M. (2014). ICA-based artefact removal and accelerated fMRI acquisition for improved resting state network imaging. NeuroImage.

[bib26] Guo C.C., Kurth F., Zhou J., Mayer E.A., Eickhoff S.B., Kramer J.H., Seeley W.W. (2012). One-year test-retest reliability of intrinsic connectivity network fMRI in older adults. NeuroImage.

[bib27] Hampson M., Driesen N., Roth J.K., Gore J.C., Constable R.T. (2010). Functional connectivity between task-positive and task-negative brain areas and its relation to working memory performance. Magn. Reson. Imaging.

[bib28] He H., Liu T.T. (2011). A geometric view of global signal confounds in resting-state functional MRI. NeuroImage.

[bib29] Ibinson J.W., Vogt K.M., Taylor K.B., Dua S.B., Becker C.J., Loggia M., Wasan A.D. (2015). Optimizing and interpreting insular functional connectivity maps obtained during acute experimental pain: the effects of global signal and task paradigm regression. Brain Connect..

[bib30] Jo H.J., Gotts S.J., Reynolds R.C., Bandettini P.A., Martin A., Cox R.W., Saad Z.S. (2013). Effective preprocessing procedures virtually eliminate distance-dependent motion artifacts in resting state FMRI. J. Appl. Math..

[bib31] Keller C.J., Bickel S., Honey C.J., Groppe D.M., Entz L., Craddock R.C., Lado F.A., Kelly C., Milham M., Mehta A.D. (2013). Neurophysiological investigation of spontaneous correlated and anticorrelated fluctuations of the BOLD signal. J. Neurosci..

[bib32] Kruschwitz J.D., Meyer-Lindenberg A., Veer I.M., Wackerhagen C., Erk S., Mohnke S., PÃ¶hland L., Haddad L., Grimm O., Tost H., Romanczuk-Seiferth N., Heinz A., Walter M., Walter H. (2015). Segregation of face sensitive areas within the fusiform gyrus using global signal regression? A study on amygdala resting-state functional connectivity. Human. Brain Mapp..

[bib33] Langfelder P., Horvath S. (2007). Eigengene networks for studying the relationships between co-expression modules. BMC Syst. Biol..

[bib34] Liang X., Wang J., Yan C., Shu N., Xu K., Gong G., He Y. (2012). Effects of different correlation metrics and preprocessing factors on small-world brain functional networks: a resting-state functional MRI study. PLoS One.

[bib35] Liang Z., King J., Zhang N. (2011). Anticorrelated resting-state functional connectivity in awake rat brain. NeuroImage.

[bib36] Macey P.M., Macey K.E., Kumar R., Harper R.M. (2004). A method for removal of global effects from fMRI time series. NeuroImage.

[bib37] Marx M., Pauly K.B., Chang C. (2012). A novel approach for global noise reduction in resting-state fMRI: applecor. NeuroImage.

[bib38] Murphy K., Birn R.M., Bandettini P.A. (2013). Resting-state fMRI confounds and cleanup. NeuroImage.

[bib39] Murphy K., Birn R.M., Handwerker D.A., Jones T.B., Bandettini P.A. (2009). The impact of global signal regression on resting state correlations: are anti-correlated networks introduced?. NeuroImage.

[bib40] Perlbarg V., Bellec P., Anton J.L., Pelegrini-Issac M., Doyon J., Benali H. (2007). CORSICA: correction of structured noise in fMRI by automatic identification of ICA components. Magn. Reson Imaging.

[bib41] Power J.D., Mitra A., Laumann T.O., Snyder A.Z., Schlaggar B.L., Petersen S.E. (2014). Methods to detect, characterize, and remove motion artifact in resting state fMRI. NeuroImage.

[bib42] Qing Z., Dong Z., Li S., Zang Y., Liu D. (2013). Global signal regression has complex effects on regional homogeneity of resting state fMRI signal. Magn. Reson. Imaging.

[bib43] Raichle M.E., MacLeod A.M., Snyder A.Z., Powers W.J., Gusnard D.A., Shulman G.L. (2001). A default mode of brain function. Proc. Natl. Acad. Sci. USA.

[bib44] Saad Z.S., Gotts S.J., Murphy K., Chen G., Jo H.J., Martin A., Cox R.W. (2012). Trouble at rest: how correlation patterns and group differences become distorted after global signal regression. Brain Connect.

[bib45] Schoelvinck M.L., Maier A., Ye F.Q., Duyn J.H., Leopold D.A. (2010). Neural basis of global resting-state fMRI activity. Proc. Natl. Acad. Sci. USA.

[bib46] Schwarz A.J., McGonigle J. (2010). Negative edges and soft thresholding in complex network analysis of resting state functional connectivity data. NeuroImage.

[bib47] Shmueli K., van Gelderen P., de Zwart J.A., Horovitz S.G., Fukunaga M., Jansma J.M., Duyn J.H. (2007). Low-frequency fluctuations in the cardiac rate as a source of variance in the resting-state fMRI BOLD signal. NeuroImage.

[bib48] Sinclair B., Hansell N.K., Blokland G.A.M., Martin N.G., Thompson P.M., Breakspear M., de Zubicaray G.I., Wright M.J., McMahon K.L. (2015). Heritability of the network architecture of intrinsic brain functional connectivity. NeuroImage.

[bib49] Song J., Desphande A.S., Meier T.B., Tudorascu D.L., Vergun S., Nair V.A., Biswal B.B., Meyerand M.E., Birn R.M., Bellec P., Prabhakaran V. (2012). Age-related differences in test-retest reliability in resting-state brain functional connectivity. PLoS One.

[bib50] Spisak T., Jakab A., Kis S.A., Opposits G., Aranyi C., Beranyi E., Emri M. (2014). Voxel-wise motion artifacts in population-level whole-brain connectivity analysis of resting-state fMRI. PLoS One.

[bib51] Wang Z. (2012). Improving cerebral blood flow quantification for arterial spin labeled perfusion MRI by removing residual motion artifacts and global signal fluctuations. Magn. Reson. Imaging.

[bib52] Weissenbacher A., Kasess C., Gerstl F., Lanzenberger R., Moser E., Windischberger C. (2009). Correlations and anticorrelations in resting-state functional connectivity MRI: a quantitative comparison of preprocessing strategies. NeuroImage.

[bib53] Wise R.G., Ide K., Poulin M.J., Tracey I. (2004). Resting fluctuations in arterial carbon dioxide induce significant low frequency variations in BOLD signal. NeuroImage.

[bib54] Wong C.W., DeYoung P.N., Liu T.T. (2015). Differences in the resting-state fMRI global signal amplitude between the eyes open and eyes closed states are related to changes in EEG vigilance. NeuroImage.

[bib55] Wong C.W., Olafsson V., Tal O., Liu T.T. (2012). Anti-correlated networks, global signal regression, and the effects of caffeine in resting-state functional MRI. NeuroImage.

[bib56] Wong C.W., Olafsson V., Tal O., Liu T.T. (2013). The amplitude of the resting-state fMRI global signal is related to EEG vigilance measures. NeuroImage.

[bib57] Yan C.G., Cheung B., Kelly C., Colcombe S., Craddock R.C., Di Martino A., Li Q., Zuo X.N., Castellanos F.X., Milham M.P. (2013). A comprehensive assessment of regional variation in the impact of head micromovements on functional connectomics. NeuroImage.

[bib58] Zarahn E., Aguirre G.K., D'Esposito M. (1997). Empirical analyses of BOLD fMRI statistics. I. Spatially unsmoothed data collected under null-hypothesis conditions. NeuroImage.

[bib59] Zhang D., Snyder A.Z., Fox M.D., Sansbury M.W., Shimony J.S., Raichle M. (2008). Intrinsic functional relations between human cerebral cortex and thalamus. J. Neurophysiol..

[bib60] Zhu D.C., Tarumi T., Khan M.A., Zhang R. (2015). Vascular coupling in resting-state fMRI: evidence from multiple modalities. J. Cereb. Blood Flow. Metab..

[bib61] Zhu L., Fan Y., Zou Q., Wang J., Gao J.H., Niu Z. (2014). Temporal reliability and lateralization of the resting-state language network. PLoS One.

[bib62] Zuo X.N., Xu T., Jiang L., Yang Z., Cao X.Y., He Y., Zang Y.F., Castellanos F.X., Milham M.P. (2012). Toward reliable characterization of functional homogeneity in the human brain: preprocessing, scan duration, imaging resolution and computational space. NeuroImage.

